# Sirt1 Promotes a Thermogenic Gene Program in Bone Marrow Adipocytes: From Mice to (Wo)Men

**DOI:** 10.3389/fendo.2019.00126

**Published:** 2019-02-28

**Authors:** Hanna Artsi, Irina Gurt, Madi El-Haj, Ralph Müller, Gisela A. Kuhn, Gal Ben Shalom, Einav Cohen-Kfir, Eva Abramowitz, Leonid Kandel, Ori Safran, Rivka Dresner-Pollak

**Affiliations:** ^1^Division of Medicine, Department of Endocrinology and Metabolism, Faculty of Medicine, The Hadassah Medical Center, Hebrew University of Jerusalem, Jerusalem, Israel; ^2^Department of Orthopedics, Faculty of Medicine, The Hadassah Medical Center, Hebrew University of Jerusalem, Jerusalem, Israel; ^3^Department of Health Sciences and Technology, Institute for Biomechanics, ETH Zurich, Zurich, Switzerland

**Keywords:** sirtuin1, marrow adipose tissue, PGC1-alpha, bone marrow mesenchymal stem cells, thermogenic genes

## Abstract

Bone marrow adipose tissue (MAT) is influenced by nutritional cues, and participates in whole body energy metabolism. To investigate the role of Sirtuin1 (Sirt1), a key player in metabolism, in MAT, marrow adiposity was evaluated in inbred 5-month-old 129/Sv *Sirt1* haplo-insufficient (*Sirt1*^Δ/+^) and wild type (WT) mice. Decreased expression of the thermogenic genes: *Prdm16, Pgc1*α*, Foxc2, Dio2*, and β*3AR* was detected in whole tibiae derived from *Sirt1*^Δ/+^ compared to WT female mice. Similarly, decreased expression of *Prdm16* and *Pgc1*α was observed in primary bone marrow mesenchymal stem cell (BM-MSC) cultures obtained from *Sirt1*^Δ/+^ compared to WT female mice, suggesting a cell autonomous effect of Sirt1 in BM-MSCs. *In vitro*, Sirt1 over-expression in the mesenchymal embryonic fibroblast stem cell line C3HT101/2 increased Pgc1α and Prdm16 protein level. Similarly, pharmacologic activation of Sirt1 by SRT3025 increased *Foxc2, Pgc1*α*, Dio2, Tfam*, and *Cyc1* expression while inhibition of Sirt1 by EX527 down-regulated *UCP1* in C3HT101/2 cells. Importantly, in human femoral BM-MSCs obtained from female patients undergoing hip operations for fracture or osteoarthritis, Sirt1 activation by SRT3025 increased *PGC1*α mRNA and protein level. Blocking sclerostin, an inhibitor of the WNT pathway and a Sirt1 target, by the monoclonal humanized antibody (Sc-AbII), stimulated β*3AR, PRDM16*, and *UCP1* gene expression, and increased PGC1α protein level. These results show that Sirt1 stimulates a thermogenic gene program in marrow adipocytes in mice and humans via PGC1α activation and sclerostin inhibition. The implications of these findings to bone health, hematopoiesis and whole body energy metabolism remain to be investigated.

## Introduction

Adipose tissue consists of three main fat depots: visceral, subcutaneous, and marrow. Marrow adipose tissue (MAT) was initially thought to be metabolically inert and a filler only. Recent studies however have found that MAT responds to nutritional cues and exercise, and participates in whole body fat metabolism (1–4). Studies in mice and humans undergoing bone marrow transplantation have demonstrated trafficking of bone marrow (BM)-derived circulating progenitor cells to adipose tissue, their differentiation into subcutaneous adipocytes and increased representation in obesity (5, 6). Furthermore, MAT was shown to contribute to circulating adiponectin in mice subjected to calorie restriction and in humans undergoing anti-cancer therapy (1). Interestingly, MAT response to nutritional cues can be distinct than visceral and subcutaneous fat depots. Calorie restriction, anti-cancer therapy, type 1 diabetes mellitus and anorexia nervosa all lead to peripheral fat loss but are surprisingly associated with increased MAT volume (7). In addition, MAT volume is inversely related to bone mass and strength in postmenopausal osteoporosis, aging and glucocorticoid excess (8–11). Finally, marrow adipocytes negatively regulate hematopoiesis and support bone homing cancers (12, 13). Thus, elucidating the mechanisms that regulate MAT may reveal novel pathways that influence bone turnover, hematopoiesis and whole body energy metabolism.

Three types of adipocytes reside in fat depots: white, brown, and beige. Lipid rich white adipocytes expand with energy intake and store triglycerides. Mitochondria rich brown adipocyte arise from a muscle-like cell lineage (*Myf5*^+^) and dissipate energy as heat, while beige adipocytes (brite) emerge in white fat depots, bear characteristics of brown adipocytes, but do not originate from the *Myf5*^+^ lineage (14).

The origin of bone marrow adipocytes is still unknown, and tools to genetically manipulate it are limited. The prevailing model suggests that a self-renewing bone marrow (BM) mesenchymal stem cell (BM-MSC) exists within the bone marrow that gives rise to osteoblasts, adipocytes, chondrocytes and marrow stromal cells (15). This BM-MSC was identified as the bone marrow stromal stem cell (BMSC) that surrounds bone marrow sinusoids (16), and has *in vivo* osteogenic and adipogenic potential. The regulatory factors that are involved in BMSC commitment to the adipocyte lineage are starting to unravel. BMSCs that express the leptin receptor (LepR) have the capacity to differentiate into both adipocytes and osteoblasts, while LepR is not expressed by neither mature osteoblasts nor marrow adipocytes, suggesting that LepR in BMSCs influences lineage allocation (17). Consistently, Leptin signaling via the LepR induced by high-fat-diet failed to promote marrow adipogenesis in mice with LepR deletion in BMSCs but not in osteoblasts, confirming that the effect is restricted to BMSCs (18). Another hormonal pathway affecting the BMSC fate is the parathyroid hormone/parathyroid hormone related peptide (PTH/PTHrP) receptor signaling pathway. Genetic loss PTH/PTHrP receptor (PTH1R) in mesenchymal stem cells using the paired related homeobox transcription factor 1 (*Prx1*)*-Cre* driver was reported to induce marrow adipogenesis, while PTH administration reduced marrow fat in mice and male patients with idiopathic osteoporosis, suggesting that PTH inhibits the differentiation of adipocyte progenitors to the adipocyte lineage (19). On another level of complexity, region-specific variation in MAT development, regulation and phenotype was reported in mice, rats and humans (20).

Sirtuin1 (Sirt1), a member of the sirtuin family of NAD^+^-dependent protein deacetylases, is a key cellular energy sensor and a mediator of the beneficial effects of calorie restriction in some animal models (21). Sirt1 regulates glucose and fat metabolism (22, 23). *In vitro*, Sirt1 inhibited the generation of white adipocytes in 3T3L-1 pre-adipocytes by down-regulating Pparγ, a master gene in white adipocytes differentiation (24). *In vivo*, adipose selective over-expression of a dominant negative Sirt1 resulted in dyslipidemia and ectopic lipid deposition (25). Targeted Sirt1 deficiency in mature adipocytes accelerated the onset of obesity-induced insulin resistance and glucose intolerance (26). On the other hand, Sirt1 gain-of-function induced a brown adipocyte-like phenotype in white adipocytes by deacetylating Pparγ and modulating its transcriptional activity (27).

Others and we have previously reported that Sirt1 directly regulates bone osteoblasts, osteoclasts and osteocytes (28–33). However, the role of Sirt1 in MAT is still largely unknown. *In vitro*, Sirt1 and its pharmacologic activation decreased adipogenesis of bone marrow MSCs (34–36). MSC-specific S*irt1* knock-out mice using the *Prx1-Cre* driver (MSCKO mice) exhibited reduced subcutaneous fat with aging, but no significant change in marrow adipocyte size compared to young mice (37).

Marrow adipogenesis is influenced by the WNT signaling pathway (38, 39). We have previously reported that Sirt1 is a negative regulator of sclerostin, an inhibitor of the canonical WNT pathway in bone (28). Our findings were recently confirmed (40). Moreover, we have shown that the administration of the Sirt1 activator, SRT3025 reduced sclerostin in bone in mice *in vivo* (29), and in human femoral BM-MSCs *in vitro* (41). In the current study we investigated the role of Sirt1 in MAT, and discovered that it induces a thermogenic gene program, characteristic of brown adipocytes, in mouse and human BM-MSCs via PGC1α stimulation and sclerostin inhibition.

## Methods

### Animals

S*irt1* haplo-insufficient mice (*Sirt1*^Δ/+^) lacking exon 4 of the *Sirt1* gene and their wild type (WT) littermates of 129/Sv background were a generous gift (see Acknowledgments), and were used for this study (42). Adult 5–7-month-old inbred *Sirt1*^Δ/+^ and WT female and male mice were studied. Animals were housed under specific pathogen free (SPF) conditions with free access to water and chow #2018 (Teklad Diets, Madison WI), containing 6.2% fat and energy density of 3.1 kCal/gr. Daily food intake was determined for each mouse for 3 weeks between age 5 and 6 months. Fasting (overnight) glucose was determined in blood collected from the tail vein by an automatic glucometer (Accuchek; Roche Diagnostics GmbH, Mannheim, Germany). For Glucose Challenge Test (GCT) mice were fasted overnight. Glucose 2 g/kg was injected intra-peritoneal, and blood was collected in 15 min intervals for 2 h. Mice were sacrificed using isoflurane inhalation (Minrad INC, USA). Whole tibia with marrow and L3–L5 were removed, immediately frozen in liquid nitrogen and stored in −80°C until analyzed. For bone fat volume determination tibiae were kept in 10% formalin for 48 h and then transferred to phosphate buffered saline (PBS) until analyzed. For bone histology tibiae were dehydrated in 50% and then in 70% EtOH and kept at 4°C. All experiments were performed with the approval of the Animal Study Committee of the Hebrew University-Hadassah Medical School (MD-12-13154-3).

### Determination of Bone Marrow Fat

To determine tibiae bone marrow fat volume osmium tetroxide staining followed by micro-computed tomography (μCT) analysis was performed in WT and *Sirt1*^Δ/+^ female mice, as previously described (43). Briefly, tibiae were fixed in 10% formalin, decalcified in 0.5 M EDTA, soaked in a 1:1 solution of equal volumes of 2% aqueous osmium tetroxide (O_s_O_4_) and 5% potassium dichromate. The intact bones were scanned at 6 μm resolution using micro-focus conebeam X-ray computed tomography (μCT40 Scanco Medical AG Brüttisellen, Switzerland). The scanner was operated at 55 KVp, 144 μA, collecting 2,000 projections per rotation at 300 ms integration time. Total tibiae marrow fat volume as well as marrow fat volumes in the proximal and distal fourths were measured separately for each mouse. Histologic analysis was performed on 4 μm thick decalcified tibial sections from the proximal tibiae and stained with hematoxylin. Adipocytes were identified as empty oval structures and were manually counted, as previously described (44). Bone marrow fat area was determined as the fraction of adipocyte area per total area. Images were obtained with a DS-Fi camera attached to an Eclipse 80i microscope (Nikon, USA), and analyzed with ImageJ (National Institutes of Health, USA, https://imagej.nih.gov/ij/).

### Primary Bone Marrow Cell Cultures

Bone marrow cells were harvested from femurs and tibiae of 6 month-old *Sirt1*^Δ/+^ and WT female mice. The femurs and tibiae were removed and cleaned of connective tissue, the ends were cut, and the marrow was flushed with α-MEM/15% fetal bovine serum. Single-cell suspensions were prepared in α-MEM by drawing the cells several times through graded needles. Cells collected from each mouse were plated in 100 mm plate. Non-adherent cells were removed after 3 days and the medium was changed every 3 days. Ten days later cells were harvested and plated in a density of 2x10^5^ cells/well in six-well-plates. Adipogenesis was induced by 10 μg/ml insulin/50 μM dexamethasone/100 μM indomethacin/500 μM 3-isobutyl-1-methylxanthine administered on day 14 post plating at 70% confluence. RNA was isolated on day 3 post adipogenic induction.

### Experiments in the Murine Mesenchymal Stem-Cell Line C3H10T1/2

The C3H10T1/2 (ATCC CCL-226) murine mesenchymal stem cell line is an established cell line model to investigate bone marrow adipocytes (45, 46). Sirt1 over-expressing C3H10T1/2 cells (*Sirt1-OE*) were previously generated and reported by us through stable retroviral infection with pBABE-*Sirt1* (28). Adipogenesis was induced in C3H10T1/2 and in *Sirt1-OE* cells with 10 μg/ml insulin/50 μM dexamethasone/100 μM indomethacin/500 μM 3-isobutyl-1-methylxanthine administered for 4 days followed by 10 μg/ml insulin/50 μM dexamethasone/5 μM rosiglitazone administration with medium changes twice a week (47). Protein was purified on day 7 post adipogenic induction. Adipogenesis was determined by oil-red-o staining on day 8–10 and was normalized to cell number determined by crystal violet staining (28, 48). In another set of experiments the Sirt1 activating compound SRT3025 (29, 49), kindly provided by SIRTRIS/GSK, was dissolved in dimethyl sulfoxide (DMSO) according to the manufacturer's instructions and was co-administered at a final concentration of 10 μM with the adipogenic medium to C3H10T1/2 cells. RNA was isolated on day 1. Oil-red-o staining and protein purification were conducted as described above.

The Sirt1 inhibiting compound Ex527 (6-Chloro-2,3,4,9-tetrahydro-1H-Carbazole-1-carboxamide; E7034, Sigma-Aldrich, Ukraine) (29, 50, 51) was dissolved in dimethyl sulfoxide (DMSO) according to the manufacturer's instructions and was co-administered at a final concentration of 10 μM with the adipogenic medium to C3H10T1/2 cells. RNA purification was conducted as described above.

### Experiments in Human Bone Marrow Mesenchymal Stromal Cells

Human bone marrow mesenchymal stromal cells (hBM-MSCs) have the capacity to spontaneously differentiate into adipocytes in *ex vivo* cell cultures without the addition of an adipogenic medium (52). Fresh femoral bone marrow was harvested during femoral canal preparation from three female patients (age 68 ± 9.3 years) undergoing hip replacement for hip osteoarthritis or fractured head of femur (*n* = 4, age 81 ± 8.1), as part of an ongoing research project which was previously reported by us (41). None of the patients had diabetes or was treated with medications known to affect glucose, lipid or bone metabolism. The study was approved by the Hadassah-Hebrew University Medical Center ethics committee (HMO-0369-10), and informed consent was obtained from each patient prior to surgery. The bone marrow aspirate was collected in growing medium (GM) containing DMEM/5 mM glucose/10%FBS/100 Units/ml penicillin/100 mg/ml streptomycin sulfate/0.25 mg/ml amphotericin B, treated with Lymphoprep #1114544 (Ficoll, Axis-Shield PoC AS, Oslo, Norway), and centrifuged at 900 g for 30 min. Cells at the intermediate interface were collected and centrifuged again at 900 g for 10 min. The resulting mononuclear pellet was re-suspended in GM, plated at a density of 5 × 10^5^ cells/35 mm dish and cultured in GM with a medium change twice a week (53). The Sirt1 activator SRT3025 at concentration of 5 μM or a vehicle was added upon confluence and with every medium change. RNA was collected 3 days following treatment initiation, while protein collection was carried out on day 10.

### Gene Expression Analysis

Whole tibiae and vertebrae with marrow, primary cultures of BMSCs, C3H10T1/2 cells and human BM-MSCs were homogenized in TRIzol (Invitrogen, Carlsbad CA). Total RNA was extracted, converted to cDNA using the qScript kit (Quanta BioSciences, Inc. Gaithersburg, MD, USA). Gene expression analysis was performed using SYBR Green-based real-time-PCR (Kapa Syber, Kapa Biosystems (Pty) Ltd, Cape Town, South Africa). Supplementary Table 1 provides all of the primer sequences used in this study. Relative gene expression was determined by the comparative CT method with β*Actin* and *Polr2a* as controls in murine and cell line experiments (geometric mean). β*Actin* was used as control for analysis of experiments conducted in human BM-MSCs. For experiments conducted in C3H10T1/2 cells gene expression was further normalized to the expression of *Adipoq*. For experiments conducted in human BM-MSCs data was further normalized to *PPAR*γ expression.

### Protein Analysis

Protein was extracted in Laemmli buffer (2% SDS/10% glycerol/5% 2-mercaptoethanol/ 0.01% bromphenol blue/60 mM Tris HCl). Antibodies for immunoblotting: Prdm16 (AbCam, ab106410), Pgc1α (Cell Signaling, #2178). α-Tubulin (AbCam, ab106375). Glyceraldehyde-3-phosphate dehydrogenase (GAPDH, Abcam, ab8245).

### Statistical Analysis

Results are presented as Mean ± SEM. Data was analyzed by unpaired Student's *t-*test to compare group means. 1-way ANOVA followed by Holm-Sidak's analysis was used to compare three groups. Analysis was performed using GraphPad (San Diego, CA, USA) Prism version 6.01. Each experiment was conducted in triplicates and was repeated at least 3 times. *P* < 0.05 was considered statistically significant.

## Results

### Reduced Expression of Thermogenic Genes in MAT of Sirt1^Δ/+^ Female Mice

**Figure 1:**
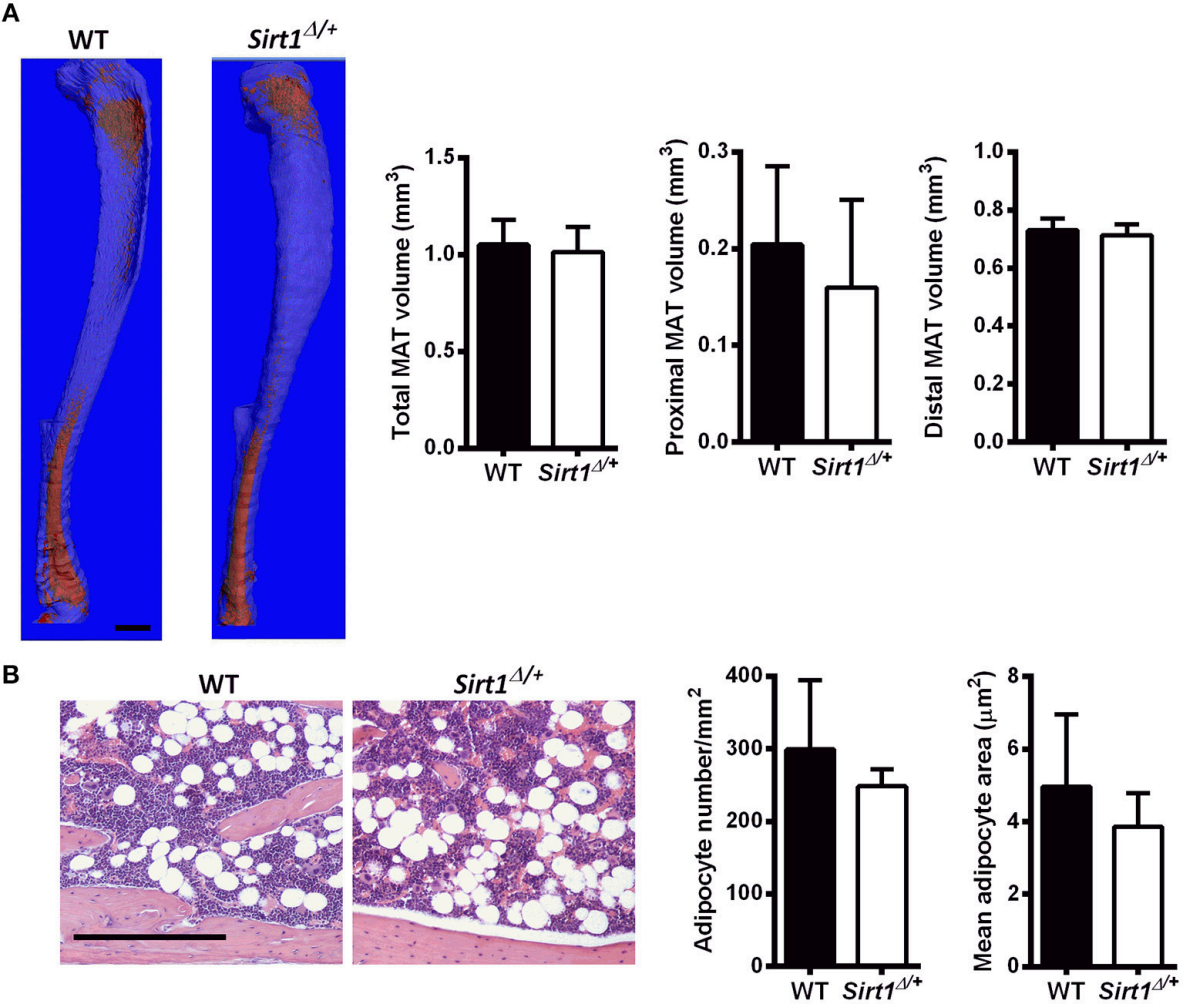
Marrow adipose tissue (MAT) in *Sirt1*^Δ/+^ and WT female mice **(A)**. Osmium tetroxide staining of tibiae followed by μCT analysis; A representative image (left) and quantification (right). Data is presented as fat volume. Scale bar 1 mm (*n* = 8 mice/group). **(B)** Hematoxylin-stained histological sections of proximal tibiae. Scale bar 200 μm; (*n* = 3 mice/group). Results are Mean ± SEM.

**Figure 2:**
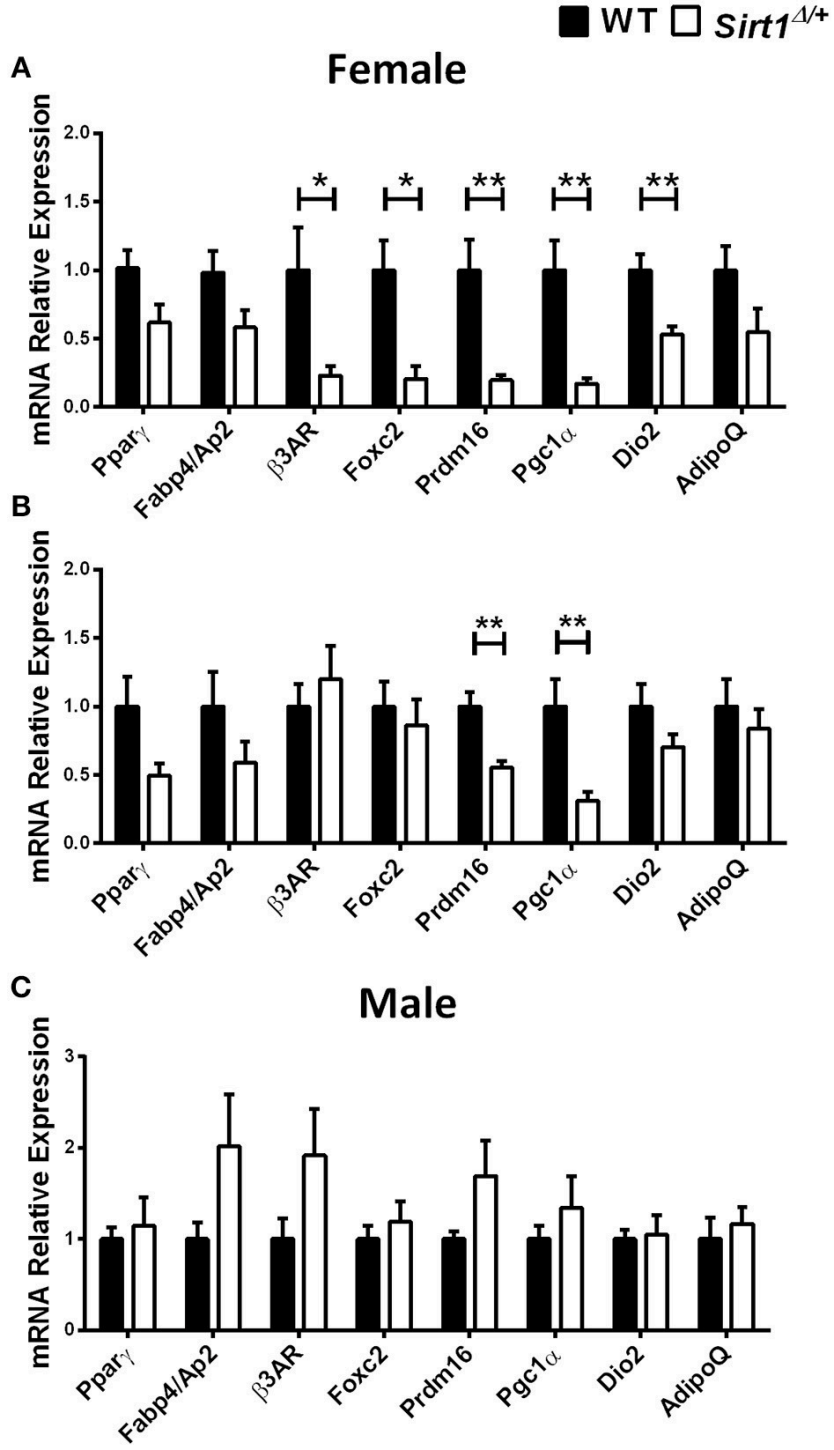
Gene expression analysis of adipocyte markers in tibial MAT and primary bone marrow stromal cell cultures obtained from *Sirt1*^Δ/+^ and WT mice **(A)**. Gene expression analysis of adipocyte markers in tibial MAT obtained from 5-month old *Sirt1*^Δ/+^ and WT female mice (*n* = 6–9 mice/group). **(B)** Gene expression analysis of adipocyte markers in primary bone marrow stromal cell cultures induced to adipogenesis, derived from *Sirt1*^Δ/+^ and WT female mice (*n* = 6 mice/group). **(C)** Gene expression analysis of adipocyte markers in tibial MAT obtained from 5-month old *Sirt1*^Δ/+^ and WT male mice (*n* = 6–9 mice/group). Results are Mean ± SEM. ^*^P < 0.05; ^**^P < 0.01 vs. wild type mice (WT).

**Figure 3:**
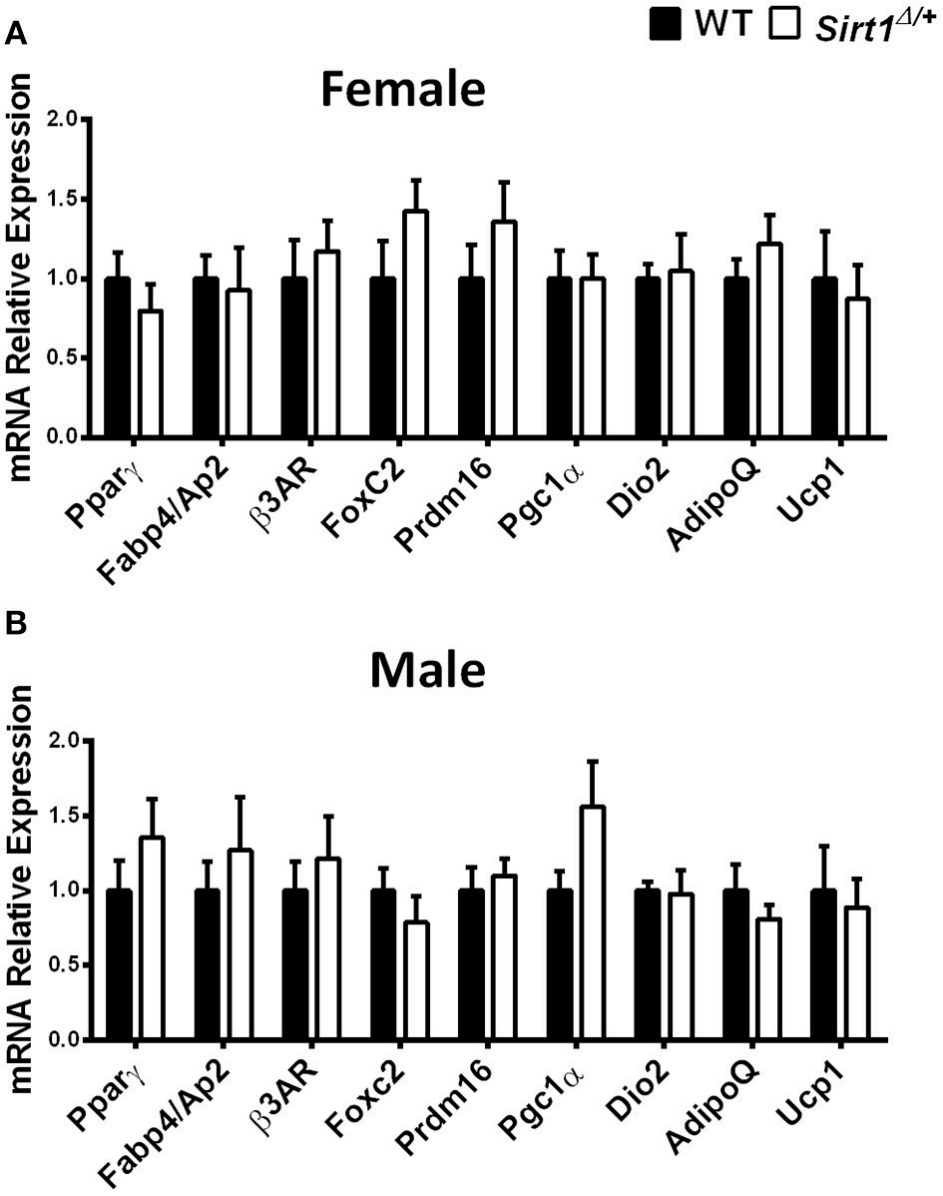
Gene expression analysis of adipocyte markers in vertebral MAT in *Sirt1*^Δ/+^ and WT female and male mice, Gene expression analysis of adipocyte markers in vertebral MAT obtained from 5-month old *Sirt1*^Δ/+^ and WT female **(A)** and male **(B)** mice (*n* = 10 mice/group). Results are Mean ± SEM.

Basal metabolic parameters including body weight, daily food intake, fasting glucose and the response to glucose load did not differ between WT and *Sirt1*^Δ/^^+^ mice of both genders (Supplementary Figures 1A–H). Previous work by others and us has demonstrated a sexual dimorphism with regard to the effects of Sirt1 deficiency in bone, showing a bone phenotype in female but not in male mice (28, 30, 31). We therefore conducted most of our studies in female mice. Whole tibiae bone marrow fat volume determined by osmium staining was not different between WT and *Sirt1*^Δ/^^+^ female mice (Figure 1A). Proximal and distal tibial marrow fat volume also did not differ between WT and *Sirt1*^Δ/^^+^ mice. In agreement with these results, tibial marrow adipocyte number and area was similar in WT and *Sirt1*^Δ/^^+^ female mice (Figure 1B). Strikingly, gene expression analysis in tibial MAT revealed a dramatic decrease of ~50% in the thermogenic genes: β*3AR, FoxC2, Prdm16, Pgc1*α, and *Dio2* in *Sirt1*^Δ/+^ compared to WT female mice (Figure 2A). Importantly, *Prdm16* and *Pgc1*α mRNA expression was significantly decreased by over 2 fold in *Sirt1*^Δ/^^+^ compared to WT-derived primary BMSC cultures induced to adipogenesis (Figure 2B), indicating a cell autonomous effect of Sirt1 on the bone marrow adipocyte. No differences in tibial MAT gene expression were observed in *Sirt1*^Δ/+^ compared to WT male mice (Figure 2C). Thermogenic genes expression in vertebral MAT did not differ between genotypes in both genders (Figures 3A,B). Taken together, these results indicate that Sirt1 haplo-insufficiency leads to reduced thermogenic genes expression in tibial MAT in female mice.

We next asked if Sirt1 stimulation reciprocally increases the expression of a thermogenic genes program in BMSCs. To address this question three *in vitro* models were employed: (1) Sirt1 over-expressing C3H10T1/2 cells induced to adipogenesis. (2) Pharmacologic activation of Sirt1 by SRT3025 in C3H10T1/2 cells induced to adipogenesis. (3) Pharmacologic activation of Sirt1 in primary human femoral BM-MSCs.

### Increased Thermogenic Markers in Sirt1 Over-Expressing C3H10T1/2 cells

**Figure 4:**
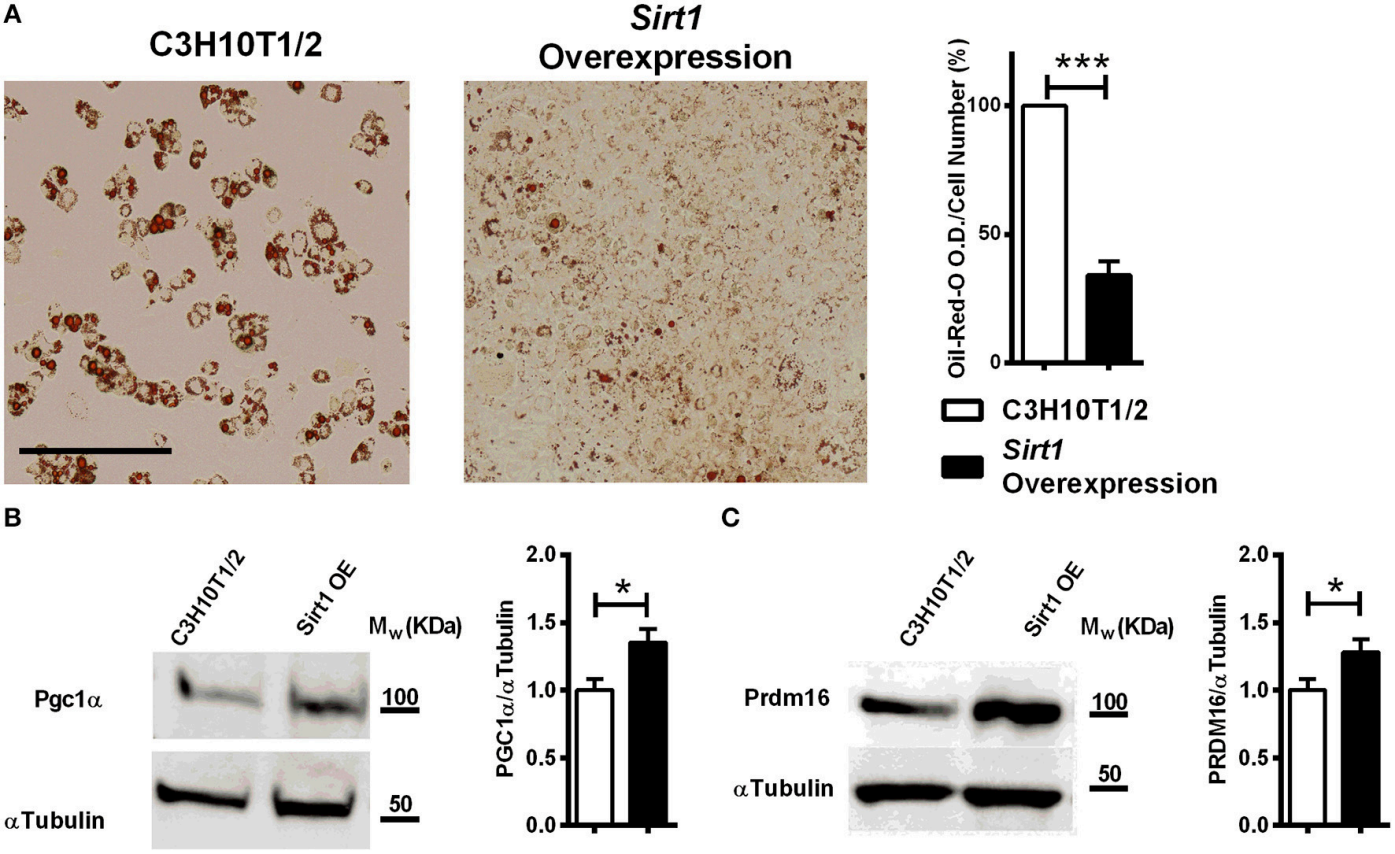
The effect of *Sirt1* over-expression on adipogenesis in C3HT101/2 cells **(A)**. Oil-red-o staining in *Sirt1* over-expressing (*OE*) and C3H10T1/2 cells induced to adipogenesis. Data is presented as optical density (OD) corrected for cell number (crystal violet staining). Scale bar 500μm. **(B,C)** Immunoblot of Pgc1α and Prdm16 in *Sirt1 OE* and C3H10T1/2 cells 7 days post induction to adipogenesis. Results are Mean ± SEM. ^*^P < 0.05; ^***^P < 0.001 vs. C3HT101/2 cells.

**Figure 5:**
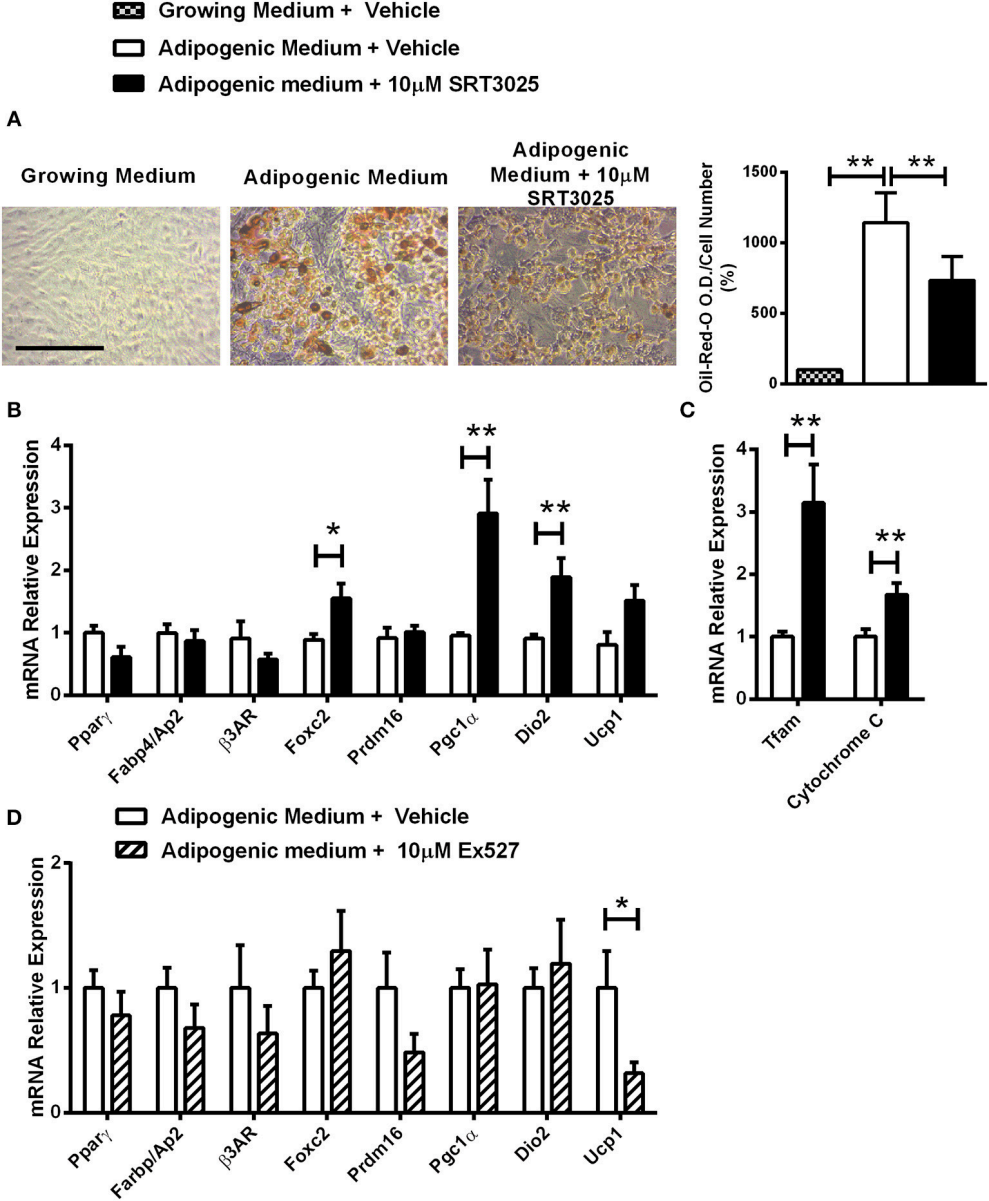
The effects of Sirt1 pharmacologic activation and inhibition on adipogenic markers in C3HT101/2 cells **(A)**. Oil-red-o staining in C3H10T1/2 cells induced to adipogenesis and supplemented with SRT3025 or vehicle (DMSO). **(B,C)** Gene expression analysis of adipocyte **(B)** and mitochondrial markers **(C)** induced to adipogenesis and supplemented with SRT3025 or vehicle (DMSO). **(D)** Gene expression analysis of adipocyte markers induced to adipogenesis and supplemented with Ex527 or vehicle (DMSO). Results are Mean ± SEM. ^*^P < 0.05; ^**^P < 0.01 vs. vehicle-treated C3HT101/2 cells.

Decreased lipid accumulation was observed in *Sirt1 OE* compared to control C3H10T1/2 cells induced to adipogenesis (Figure 4A). Elevated Prdm16 and Pgc1α protein level was observed in *Sirt1 OE* compared to control cells (Figures 4B,C). Consistent with these results, pharmacologic activation of Sirt1 by SRT3025 reduced the generation of white adipocytes and stimulated the expression of *Pgc1*α*, Dio2, FoxC2, Tfam*, and *Cytochrome C* (Figures 5A–C). In contrast, Sirt1 inhibition by Ex527 significantly reduced the expression of *Ucp1* (Figure 5D).

### Sirt1 Activation by SRT3025 Promotes Thermogenic Genes Expression in Human BM-MSCs

**Figure 6:**
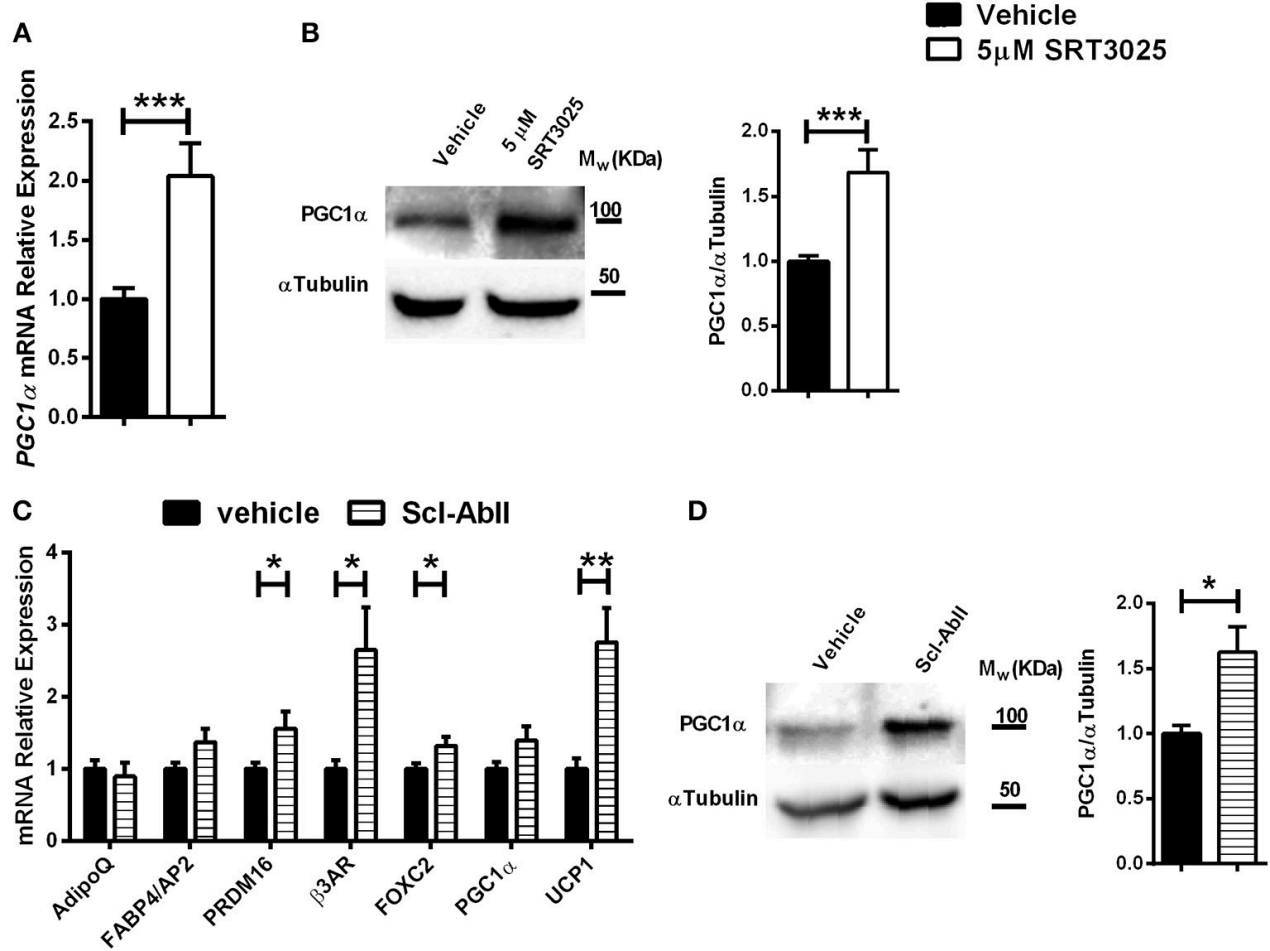
The effects of SRT3025 and anti-Sclerostin antibody on adipogenic markers in human femoral bone marrow mesynchemal stem cells (hBM-MSCs) **(A)**. Gene expression analysis of PGC1α in SRT3025-treated hBM-MSCs. **(B)** Immunoblot of PGC1α. A representative image (left) and densitometry (right) with αTubulin as control. **(C)** Gene expression analysis of thermogenic genes in anti-Sclerostin AbII treated hBM-MSCs **(D)**. Immunoblot of PGC1α in hBM-MSCs treated with anti-Sclerostin AbII. A representative image (left) and densitometry (right) with αTubulin as control. Results are mean ± SEM. ^*^P < 0.05; ^**^P < 0.01; ^***^P < 0.001 vs. vehicle-treated hBM-MSCs.

To investigate if the effects of Sirt1 on the marrow adipocyte phenotype can be extended to human BM-MSCs, SRT3025 was administered to primary human femoral BM-MSCs. Strikingly, SRT3025-treated human BM-MSCs had increased mRNA expression of *PGC1*α (Figure 6A) accompanied by elevated PGC1α protein level (Figure 6B), suggesting that Sirt1 activates PGC1α in human femoral BM-MSCs.

### Blocking Sclerostin Induces a Thermogenic Gene Program in Human BM-MSCs

We have previously shown that Sirt1 down-regulates sclersotin in mice and human BM-MSCs (28, 29, 41). As sclersotin stimulates marrow adipogenesis (54), we hypothesized that blocking sclerostin will affect human bone marrow adipocyte gene expression profile.

Indeed, blocking sclerostin with the monoclonal antibody Scl-AbII (55, 56) induced a marked increase in gene expression of: *PRDM16*, β*3AR, FOXC2*, and importantly an over 2-fold increase in *UCP1* expression, the hallmark of brown and beige adipocytes (Figure 6C). These effects were accompanied by a 2-fold increase in PGC1α protein level (Figure 6D). Taken together, this data indicate that blocking sclerostin stimulates a thermogenic genes signature in human femoral BM-MSCs.

## Discussion

This study reports for the first time a role for Sirt1 in MAT phenotype, demonstrating its stimulatory effect on a thermogenic gene program in marrow adipocytes. *Sirt1* haplo-insufficiency resulted in decreased expression of thermogenic gene markers in MAT in a gender- and site-specific manner. Reduced expression of β*3AR, FoxC2, Prdm16, Pgc1*α, and *Dio2* was found in tibial but not in vertebral MAT derived from adult female but not male *Sirt1*^Δ/^^+^ mice. Similar effects were noted in primary BM-MSC cultures derived from *Sirt1*^Δ/^^+^ mice, indicating a cell autonomous effect of Sirt1 on the BM-MSC. *In vitro*, Sirt1 over-expression in CH310T1/2 cells increased the expression of thermogenic markers characteristic of brown adipocytes Prdm16 and Pgc1α, a mitochondrial biogenesis inducer, and a known Sirt1 target (57). Importantly, in human femoral BM-MSCs pharmacologic Sirt1 activation and blocking its target, sclerostin, had a stimulatory effect on Pgc1α protein level and thermogenic genes expression.

Sirt1 was previously shown to directly deacetylase Pgc1α in liver and muscle, thereby promoting its phosphorylation by 5' adenosine monophosphate-activated protein kinase (AMPK), resulting in its activation (58, 59). The current study demonstrates for the first time that Sirt1 upregulates Pgc1α in murine and human BM-MSCs. Pgc1α was reported to increase thermogenic genes expression in white subcutaneous adipocytes (60) and brown adipocytes (61). Furthermore, Pgc1α is a master mitochondrial regulator, stimulating mitochondrial biogenesis and inducing the expression of components of the mitochondrial respiratory chain. Indeed, in our study gene expression of *Tfam*, a marker of mitochondrial biogenesis, and *Cytochrome C* was significantly increased with Sirt1 pharmacologic activation *in vitro*. The role of Pgc1α in bone and BM-MSCs was only recently revealed. *In vivo* and *in vitro* gain and loss-of-function studies demonstrated that Pgc1α regulates the skeletal stem cell fate, restraining marrow adipocyte differentiation and promoting osteogenesis (62). Our data indicate that Sirt1 stimulates Pgc1α expression in BM-MSCs thereby leading to induction of a thermogenic gene program.

Interestingly, Prdm16 was also consistently influenced by Sirt1 status in the various *in vivo* and *in vitro* models employed in this study. Prdm16 was significantly decreased in tibial MAT and in primary BM-MSC cultures derived from *Sirt1*^Δ/+^ compared to WT female mice. Along these lines, it was markedly increased with Sirt1 over-expression or activation *in vitro*. Prdm16 is an activator of Pgc1α expression and transcriptional function through direct protein interaction. It also induces *Pgc1*α, *Ucp1*, and *Dio2* expression in adipocytes (63). However, regulation of Prdm16 by Sirt1 has not been described before. Only one study demonstrated that Sirt1-dependent Pparγ deacetylation allows Prdm16 recruitment to Pparγ thereby modulating its transcriptional activity, favoring BAT genes expression while repressing WAT genes (27). Thus, the underlying mechanisms governing the observed changes in Prdm16 in this study remain to be elucidated.

We have previously reported that Sirt1 down-regulates sclerostin by deacetylating histones 3 and 4 at its promoter, leading to inhibition of *Sost* gene expression (28). These results were recently confirmed by Stegen et al who demonstrated that conditional deletion of the oxygen sensor prolyl hydroxylase (PHD) 2 in osteocytes resulted in enhanced HIF-1α signaling that stimulated Sirt1-dependent deacetylation of the *Sost* promoter and reduced sclerostin expression (40). Sclerostin, an inhibitor of the WNT/β-catenin pathway, was reported to induce adipocyte differentiation in 3T3-L1 cells (64), primary murine BM-MSCs (54), and human BM-MSCs (54). Along these lines, lower levels of MAT were found in tibiae of sclerostin knock-out (SOST-KO) mice, while sclerostin neutralization with a neutralizing antibody significantly decreased MAT (54). Our results demonstrate for the first time that blocking sclerostin with a neutralizing antibody, currently under advanced investigation for the treatment of osteoporosis, induces a thermogenic gene program in human BM-MSCs, and increases the expression of *UCP1*, a driver of mitochondrial heat generation and energy expenditure. UCP1, a protein located on the inner mitochondrial membrane, uncouples electron transport from adenosine triphosphate (ATP) generation. The resulting energy derived from substrate oxidation is dissipated as heat. UCP1 is expressed in brown and beige adipocytes. Some previous studies failed to detect it in MAT, while others reported low expression levels (11, 44). The metabolic significance of inducing UCP1 in MAT by blocking sclerostin remains to be investigated.

Differences in MAT phenotype between *Sirt1*^Δ/^^+^ and WT female mice were detected in tibiae but not in lumbar MAT. Lumbar vertebrae is a skeletal site in the mouse that has little MAT, whereas proximal tibial MAT was shown to be metabolically responsive to cold exposure (20). Caudal vertebrae was previously shown to have characteristics of constitutive MAT (cMAT) that contains large adipocytes and does not respond to systemic challenges. Thus, lack of difference in vertebral MAT phenotype is not surprising. Of note, most of the studies investigating murine MAT have used the C57BL/6J and C3H/HeJ mouse strains, whereas data in 129/Sv used in this study, is lacking.

This study is not without its limitations. The physiologic significance of our findings could not be evaluated in the mouse model used in this study. Exposure of MSC-specific S*irt1* knock-out mice to cold temperature or a high fat diet could have provided insight into the contribution of MAT Sirt1 to local and whole body energy metabolism and needs to be performed in future studies. Secondly, gene expression analyses were performed in whole tibiae and vertebrae extracts similar to previously published studies (4, 65), and introduce the bias of contamination by other cell types. However, consistent results were obtained in primary BM-MSCs cultures derived from *Sirt1*^Δ/+^ and WT mice, supporting the notion of a direct cell autonomous effect of Sirt1 on the marrow adipocyte phenotype. Thirdly, we did not account for regional differences in tibial MAT composition, as was previously suggested (66). Finally, additional Sirt1 targets beyond Pgc1α and sclerostin may have played a role in driving a brown-like adipocyte gene expression program in BM-MSCs.

Whether inducing a thermogenic gene program in marrow adipocytes is beneficial to bone health, hematopoiesis local and whole body energy metabolism begs further investigation. Reduced MAT expression of brown adipocyte markers was previously reported in diabetic and aged mice, conditions associated with both increased skeletal fragility and impaired energy metabolism (44). Due to its wide favorable physiologic effects, Sirt1 has been considered an attractive therapeutic target for drug discovery. Sirtuin1 activating compounds (STACs) were generated, amongst them SRT3025, used in this study. SRT3025 was previously shown by us to restore bone mass and strength in OVX mice (29), but also to have off-target effects (67). In humans SRT3025 was shown to prolong QTc and its development was discontinued (68). As NAD^+^ is an indispensable co-substrate required for Sirt1 and other sirtuins activity, there has been an increasing interest in small molecules that raise NAD+ levels as a mechanism to stimulate sirtuins activity (69).

In conclusion, this study shows that Sirt1 regulates the bone marrow adipocyte phenotype inducing a thermogenic gene program in mouse and human BM-MSCs. Inducing BAT-like features in subcutaneous and visceral fat depots is a much desired goal in combating obesity. Whether browning of MAT by Sirt1 activation, sclerostin inhibition or other mechanisms is a plausible novel approach to serve this goal while improving skeletal health remains to be elucidated.

## Ethics Statement

This study was carried out in accordance with the recommendations of IHC-GCP, Public Heath Regulation, the Governing regulations of Ministry of Health. The protocol was approved by The Ethics (Helsinki) Committee at Hadassah University Hospital. All subjects gave written informed consent in accordance with the Declaration of Helsinki.

## Author Contributions

HA designed and performed most of the experiments and analyzed the data. IG designed and conducted some of the *in vitro* experiments. ME-H, LK, and OS were responsible for studies in humans. ME-H obtained the human samples and performed the experiments. RM and GK designed and conducted the marrow fat μCT quantification studies. GB performed experiments in murine BM-MSCs. EC-K designed and performed the experiments in human BM-MSCs. EA performed some *in vitro* experiments with SRT3025. RD-P conceived and designed the study, prepared the manuscript, and takes full responsibility for the work as a whole.

### Conflict of Interest Statement

The authors declare that the research was conducted in the absence of any commercial or financial relationships that could be construed as a potential conflict of interest.

## Supplementary Material

Supplementary Figure 1 Metabolic parameters in female and male *Sirt1*^Δ/+^ and WT mice **(A–D)**: females; **(E-H)**: males. **(A,E)**: Body weight. **(B,F)**: Daily food intake. **(C,G)**. Fasting blood glucose. **(D,H)**. Glucose challenge test (GCT) Results are Mean ± SEM (*n* = 5–8 mice/group).

Supplementary Table 1 Mouse and human primer sequences.
