# Supplementary material for: Sirt1 Promotes a Thermogenic Gene Program in Bone Marrow Adipocytes: From Mice to (Wo)Men
Source: Front Endocrinol (Lausanne). 2019 Feb 28;10:126. doi: 10.3389/fendo.2019.00126 (PMC6403178; doi:10.3389/fendo.2019.00126)
Supplement: Supplementary Table 1 — Mouse and human primer sequences. [file Table_1.DOCX]

Primers for qPCR.

| **Gene name** |  | **Forward** | **Reverse** |
| --- | --- | --- | --- |
| **Pparγ** | **Mouse** | GAGATTCTCCTGTTGACCCAGA | GGAGAGGTCCACAGAGCTGAT |
| **Ap2/Fabp4** | **Mouse** | GGATGGAAAGTCGACCACAA | AGGCCTCTTCCTTTGGCTC |
| **b3AR** | **Mouse** | ACCGCTCAACAGGTTTGATG | GTCCTGCAAAAACGGAAACA |
| **Foxc2** | **Mouse** | ATCCGCAGCTCAGTTTGAGA | CATCTGGGTAGGGGAAGGAA |
| **Prdm16** | **Mouse** | GGCTCAAGGAGGAGGAGAGA | GTCCGGGTCAGGTTCATACA |
| **Pgc1α** | **Mouse** | CCAAGACTCTGTATGGAGTGACATAGA- | AAGCTGTCTGTATCCAAGTCATTCAC- |
| **Dio2** | **Mouse** | TGGAACAGCTTCCTCCTAGATG | CGAGGCATAATTGTTACCTGATTC |
| **Ucp1** | **Mouse** | GGATGGTGAACCCGACAACT | CCTGGCCTTCACCTTGGAT |
| **Adipoq** | **Mouse** | GATGGCACTCCTGGAGAGAA | TCTCCAGGCTCTCCTTTCCT |
| **Tfam** | **Mouse** | CAAAGGATGATTCGGCTCAG | AAGCTGAATATATGCCTGCTTTTC |
| **Cyc1** | **Mouse** | CAGAAGTCTTGGAGTATGATG | ATCAACAACATCTTGAGACC |
| **βactin** | **Mouse** | GATATCGCTGCGCTGGTC | GACCCATTCCCACCATCA |
| ***Polr2a*** | **Mouse** | GAAGCTGGTCCTTCGAATCC | ACTCAATGCATCGCAGGAAG |
| **PPARγ** | **Human** | GAACAGATCCAGTGGTTGCAGAT | GAGATGCAGGCTCCACTTTGAT |
| **AP2/**  **FABP4** | **Human** | CTGGGCCAGGAATTTGACGAA | TTCCATCCCATTTCTGCACATGT |
| **b3AR** | **Human** | ATGAGACCTTAGTGTTCTCC | CATTCCATGGCTAAAGTGAG |
| **FOXC2** | **Human** | CGTGCGGGAGATGTTCAACT | ATAGAGAGGCGGCGTGGAT |
| **PRDM16** | **Human** | TCTACAGCAGGGTAGAAAAG | TCTCTGTCATGGTCTCTATG |
| **PGC1a** | **Human** | CTCAAGTGGTGCAGTGACCAA | TCTGTGAGGACTGCTAGCAAG |
| **UCP1** | **Human** | CTGGAATAGCGGCGTGCTT | AATAACACTGGACGTCGGGC |
| **AdipoQ** | **Human** | CATCTGGCTAAATGGACATC | CTGAGTGATTGGTCAGAAAC- |
| **TFAM** | **Human** | GAAAGATTCCAAGAAGCTAAGG | GAGTCAGACAGATTTTTCCAG |
| **CYC1** | **Human** | CACAGATGTCTTAGAGTTTGAC | GAGCCATCATCATCAACATC |
| **βActin** | **Human** | GAGCTACGAGCTGCCTGACG | GTAGTTTCGTGGATGCCACAG |
